# Long-term effects of prior diets, dietary transition and pregnancy on adipose gene expression in dairy heifers

**DOI:** 10.1371/journal.pone.0218723

**Published:** 2019-07-03

**Authors:** Hilde K. L. Wærp, Sinéad M. Waters, Matthew S. McCabe, Paul Cormican, Ragnar Salte

**Affiliations:** 1 Department of Animal and Aquacultural Sciences, Faculty of Biosciences, Norwegian University of Life Sciences, Ås, Norway; 2 Animal and Bioscience Research Department, Animal and Grassland Research and Innovation Centre, Teagasc, Dunsany, County Meath, Ireland; Weill Cornell Medical College in Qatar, QATAR

## Abstract

Adipose tissue is highly involved in whole-body metabolism and is the main site for lipid synthesis, storage and mobilization in ruminants. Therefore, knowledge about adipose tissue responses to different diets is important, especially in growing heifers as the feeding regimes of replacement heifers affect their future success as dairy cows. However, at gene expression level such knowledge is limited. As part of a larger feed trial, adipose tissue biopsies from 24 Norwegian Red heifers were collected at 12 months of age (12MO) and at month seven of gestation (PREG) and analyzed by next-generation mRNA sequencing. Between these two sampling points, all heifers had gone through a successful conception and a feed change from four dietary treatments of high or low energy (HE/LE) and protein (HP/LP) content (treatments LPHE, HPHE, LPLE and HPLE) to a low-energy, low-protein pregnancy feed given to all animals. Gene expression differences between different feed treatments at 12MO are described in an earlier publication from our group. The main objectives of this study were to investigate the long-term effects of diets differing in protein and energy density level on gene expression in adipose tissue of growing replacement dairy heifers. To achieve this, we examined the post-treatment effects between the treatment groups at month seven of gestation; 6 months after the termination of experimental feeding, and the long-term gene expression changes occurring in the adipose tissue between 12MO and PREG. Post-treatment group comparisons showed evidence of long-term effects of dietary treatment on adipose gene expression. Differences between protein treatments were smaller than between energy treatments. Adipose gene expression changes from 12MO to PREG were much larger for the HE than the LE treatments and seemed to mostly be explained by the characteristics of the diet change. 97 genes displayed a unidirectional expression change for all groups from 12MO to PREG, and are considered to be treatment-independent, possibly caused by pregnancy or increased age. This study provides candidate genes and key regulators for further studies on pregnancy preservation (TGFB1, *CFD*) and metabolic regulation and efficiency (PI3K, RICTOR, MAP4K4,) in dairy cattle.

## Introduction

-The heifer is the cow of the future. Through the years, this recognition has brought about a plethora of heifer rearing studies investigating the relationships between heifer rearing strategies and animal performance including growth [1], mammary development [2–5], endocrinology [6], metabolic profile [7] and subsequent milk yield [7, 8]. The importance of a well-functioning metabolic regulation and an appropriate nutrient allocation in a successful dairy cow management is commonly known, and have been subject of intense research down to the level of hepatic [9–15], mammary [16] and adipose gene expression [17–23] in adult cows.

However, if one acknowledges the fact that a cow is not just a function of its genome and its environment at a given moment, but rather a function of its genome and all its previous environments, it is clear that if we are to optimize dairy management, we also need information about metabolic regulatory mechanisms at earlier life stages. In this regard, adipose tissue plays a key role as it is not only the end point for feed excess energy and an energy reserve for periods of higher energy consumption than input [24], but is also a transcriptionally and endocrinologically active organ strongly involved in the regulation of whole-body metabolism [25, 26]. There are several published gene expression studies on adipose tissue in growing cattle [23, 27–31], but so far no reports of post-treatment dietary effects or effects of pregnancy on the total adipose transcriptome. As part of a larger feed trial, adipose tissue biopsies were collected at 12 months of age (12MO) and at month seven of gestation (PREG, 20 (HE) and 24 (LE) months of age) from 24 Norwegian Red heifers and analyzed by next-generation RNA sequencing (RNA-seq). Between these two sampling points, all heifers had gone through a successful conception and a feed change from four dietary treatments differing in energy and protein content, to a low-energy, low-protein pregnancy feed given to all animals. During pregnancy, we observed different growth responses to the pregnancy feed between the dietary treatment groups. Such post-treatment differences have been described by others, and have been found to account for a larger fraction of milk yield variability than the experimental feeding per se [32]. It is therefore of great interest to investigate the metabolic regulation underlying these post-treatment effects. Thus, the main objective of this study was to examine post-treatment gene expression differences between the experimental feeding groups at PREG; six months after change to a consistent feeding regime. A secondary objective of this study was to describe the gene expression changes occurring in the heifers between 12MO and PREG. Finally, differentially expressed genes associated with pregnancy, diet change or post-treatment dietary effects identified in this study could potentially yield candidate genes for further studies on breeding and feeding for improved feed efficiency, production and reproduction in Norwegian Red cattle.

## Materials and methods

### Ethics statement

All experimental procedures involving animals were approved by the Norwegian Animal Research Authority (FOTS id 2955, reference no. 2010/203231). Biopsies were harvested under local anesthesia, and all animals were given 3 mg/ kg BW of Ketoprofen intramuscularly (Comforion vet., Orion Pharma Animal Health) to prevent pain or inflammation at the biopsy site.

### Animal model

Eighty Norwegian Red heifers from the university herd (born in 2010 and 2011) were randomly assigned either to a high (HE) or low (LE) energy group, destined for a BW gain of 850–1000 or 600–750 g/day from three months of age to confirmed pregnancy, respectively. Each of the energy groups were split into two protein groups, low (LP) or high (HP), to give four dietary treatment groups with 20 animals in each group, *viz* Low-protein high-energy (LPHE), high-protein high-energy (HPHE), low-protein low-energy (LPLE) and high-protein low-energy (HPLE). Target body weight at conception was 400 kg for all heifers. To achieve this, first insemination was carried out at first heat after passing 370 kg for the HE heifers (fast-growing) and after passing 380 kg for the LE heifers (slower-growing). No hormone treatments or other fertility enhancing treatments were applied. The aim of a common BW for all heifers at conception also meant heifers on HE treatments were inseminated at an earlier age than LE heifers, because of their faster growth. Age at successful insemination was 13.6 months for LPHE, 12.8 months for HPHE, 17.1 months for LPLE and 16.9 months for LPHE heifers, and PREG samples were taken 7 months after these age points. After confirmed pregnancy (at day 28–42 of gestation), all heifers were group-fed the same diet *ad libitum*. The pregnancy diet was designed for a moderate growth of about 550 g/ d until calving to reach a target BW of 560 kg and a maximum BCS of 3.75 at calving. Nutrient content in the experimental diets at 12 months of age and in the pregnancy diet at month seven of gestation is shown in Table 1.

**Table 1:** Average nutrient content of experimental diets at 12 months and common diet offered during pregnancy.

	12MO				PREG
	LPHE	HPHE	LPLE	HPLE	
Net energy (MJ/kg DM)	6.43	6.43	5.98	5.98	5.66
Crude protein (g/kg DM)	142	149	113	122	113
Neutral detergent fiber (g/kg DM)	508	510	579	581	569
Starch (g/kg DM)	40	26	49	32	0

Experimental diets were Low-protein, High-energy (LPHE), High-protein, High-energy (HPHE), Low-protein, Low-energy (LPLE) and High-protein, Low-energy (HPLE). PREG = Pregnancy diet given to all animals. Total ration content for 12MO diets was calculated in Optifôr, a digital feed planning tool based on NorFôr, the Nordic feed evaluation system for cattle [33]. Input data were the nutrient content of the roughages and concentrates used and least square means of roughage and concentrate intake at 12 months for each treatment. Total ration content for the PREG diet was calculated as the average nutrient content of the roughages offered during pregnancy.

### Adipose biopsy sampling and sample preparation

Adipose tissue biopsies were harvested at 12 months of age (12MO) and at month seven of gestation (PREG). These time points were chosen to assess the long-term between-treatment differences and within-treatment changes when the heifers were in a steady state, without the interference of a recent feed change or an imminent calving.

Biopsy sampling procedure, RNA extraction and library preparation were performed as described by Wærp et al. [34]. Briefly, 0.5–1.5 g of adipose tissue was harvested from the tail base of the heifers under epidural anesthesia. Biopsies were snap frozen in liquid nitrogen, and subsequently stored at -80°C. 48 Adipose tissue samples from 24 heifers were selected for RNA sequencing (n = 6 for each group). RNA extraction was carried out using the Qiagen RNeasy lipid tissue mini kit (Qiagen). The quality and RIN values of the extracted samples were assessed using an Agilent bioanalyzer. RIN values varied between 7.8 and 9.2. To avoid DNA contamination, all RNA samples were subject to a DNAse treatment after extraction (Turbo DNA-free, Ambion, Life technologies) and a subsequent cleanup (RNA Clean & Concentrator -25, Zymo Research Corp).

Library preparation was performed using the Illumina TruSeq RNA sample prep kit v2, according to the manufacturer’s manual.Sequencing was performed in an Illumina HiSeq 2000 workstation, with 100 bp paired end reads, and four samples per lane (Clinical Genomics, Toronto).

### Statistics and Bioinformatics

The fastq files containing the raw sequence reads were examined using FASTQC (v 0.11.2.) Adapters were trimmed from all sample fastq files using Cutadapt (v 1.3). Reads were simultaneously quality trimmed by removing reads shorter than 20 bp, or with Phred scores below 25. Following adapter and quality trimming, all files were checked again in FASTQC. Paired-end read files were aligned to the UMD3.1 bovine genome assembly [35], using Tophat (v 2.0.12) [36]. The number of reads per gene in each sample was counted using HTSeq-count (v 0.6.1) [37]. Statistical analysis of read counts were carried out in EdgeR (v 3.1.2), a Bioconductor software package run in the statistical software environment R (v 3.1.2) [38]. All differential gene expression analyses were performed in EdgeR, using tagwise dispersions. The statistical tests were corrected for multiple testing using the Benjamini-Hochberg method as implemented in EdgeR [39, 40]. To compare gene expression differences between treatments, a generalized linear model which included dietary treatment and genetic group was fitted to the data using the glmFit function [39]. As EdgeR cannot estimate multiple levels of variation, the 12MO and PREG sample sets were divided into subsets and analyzed for treatment differences in separate runs, to avoid bias caused by unaccounted within-individual variation (due to the paired nature of the total data set).

The comparisons were performed as pairwise comparisons between contrasting treatments, i.e. LPHE-LPLE, HPHE-HPLE, LPHE-HPHE and LPLE-HPLE. Significantly differentially expressed genes (DEG) were called at a FDR (false discovery rate adjusted p-value as given in EdgeR) of 0.05. A full description of the DEG representing treatment differences at 12 months of age (12MO samples), is presented in a separate paper by our group [34]. To correct for any effect of individual animals, the difference in gene expression between 12MO and PREG was analyzed as a pairwise comparison within each treatment group. Subsequently, the resulting DEG lists were compared to find the DEG common to all treatments. Gene expression changes from 12MO to PREG which were common to all treatments was assumed to have occurred independently of previous dietary treatment, and to rather be caused by pregnancy or increased age. The lists of differentially expressed genes from each comparison were subsequently analyzed through the use of IPA (QIAGEN Inc., https://www.qiagenbioinformatics.com/products/ingenuity-pathway-analysis/ [41], to find over-represented biological pathways and upstream regulators represented by the DEG. Significance level for the pathway analyses was set at p < 0.05. Finally, we run an IPA comparison analysis of the 12MO-PREG DEG lists within each treatment was to compare the gene expression changes between 12MO and PREG and the upstream regulators controlling them for each dietary treatment. Pathways or functions specifically pertaining to irrelevant diseases, species or tissues were omitted. The RNAseq raw data are deposited and accessible through NCBI’s Gene Expression Omnibus with the GEO Series accession number GSE79347.

## Results and discussion

### Animal performance

Body weight and BCS at 12 months of age, conception, 7 months of pregnancy and calving are shown in Table 2. Both HE and LE heifers followed their planned growth trajectories throughout the experimental feeding period, with an average daily gain (ADG) of 930 and 660 g/d, respectively. Body weight at conception was close to 400 kg for all treatment groups, as shown in Table 2. The study design, aiming for a similar body weight at conception for all heifers, led to the slower-growing LE heifers being about 4 months older than the HE heifers at time of conception (Table 2). The response to the pregnancy diet differed between treatment groups: LE heifers displayed a faster growth than expected with an ADG of 600 g/d, while the HE heifers only achieved an ADG of 470 g/d during pregnancy. This is thought to be caused by a late compensatory growth effect occurring in the LE heifers, which were restrictively fed during the experimental feeding period, while all heifers were fed ad lib during pregnancy. This led to the HE heifers calving in at a BW of approximately 535 kg at 22 months of age, while the LE heifers calved in at 26 months at a BW of approximately 565 kg. The different pregnancy growth rates were not planned, and thus represents a complicating factor which must be taken into account in the interpretation of our results.

**Table 2:** Least square means of body weight (BW) and body condition score (BCS) at 12 months of age, conception, 7 months of pregnancy and calving.

	Treatment	Age (mo)	BW (kg)	BCS (scale 1–5)
12 months	LPHE	12	363 (0.9)^a^	3.99 (0.016)^a^
	HPHE	12	371 (1.1)^b^	3.95 (0.017)^a^
	LPLE	12	295 (0.8)^c^	3.63 (0.014)^b^
	HPLE	12	311 (0.8)^d^	3.63 (0.013)^b^
Conception	LPHE	13.6	411 (5.2)^a^	3.98 (0.02)
	HPHE	12.8	396 (5.3)^ab^	3.93 (0.02)
	LPLE	17.1	393 (5.2)^b^	3.73 (0.02)
	HPLE	16.9	406 (5.2)^ab^	3.73 (0.02)
7 months pregnant	LPHE	20.6	483 (3.8)^a^	3.80 (0.010)^a^
	HPHE	19.8	477 (3.7)^a^	3.79 (0.010)^a^
	LPLE	24.1	506 (3.9)^b^	3.75 (0.011)^b^
	HPLE	23.9	516 (3.9)^c^	3.74 (0.011)^b^
Calving	LPHE	22.5	537 (11.4)^a^	3.70 (0.02)
	HPHE	21.9	532 (10.7)^a^	3.65 (0.02)
	LPLE	26.1	571 (11.0)^b^	3.71 (0.02)
	HPLE	25.8	564 (11.7)^a^	3.73 (0.02)

LPHE = Low-protein, High-energy; HPHE = High-protein, High-energy; LPLE = Low-protein, Low-energy and HPLE = High-protein, Low-energy. N = 80 (all animals in the feeding experiment). Numbers with different superscripts differ. Standard errors are presented in parentheses.

### Read alignment and overall results

**Fig 1:**
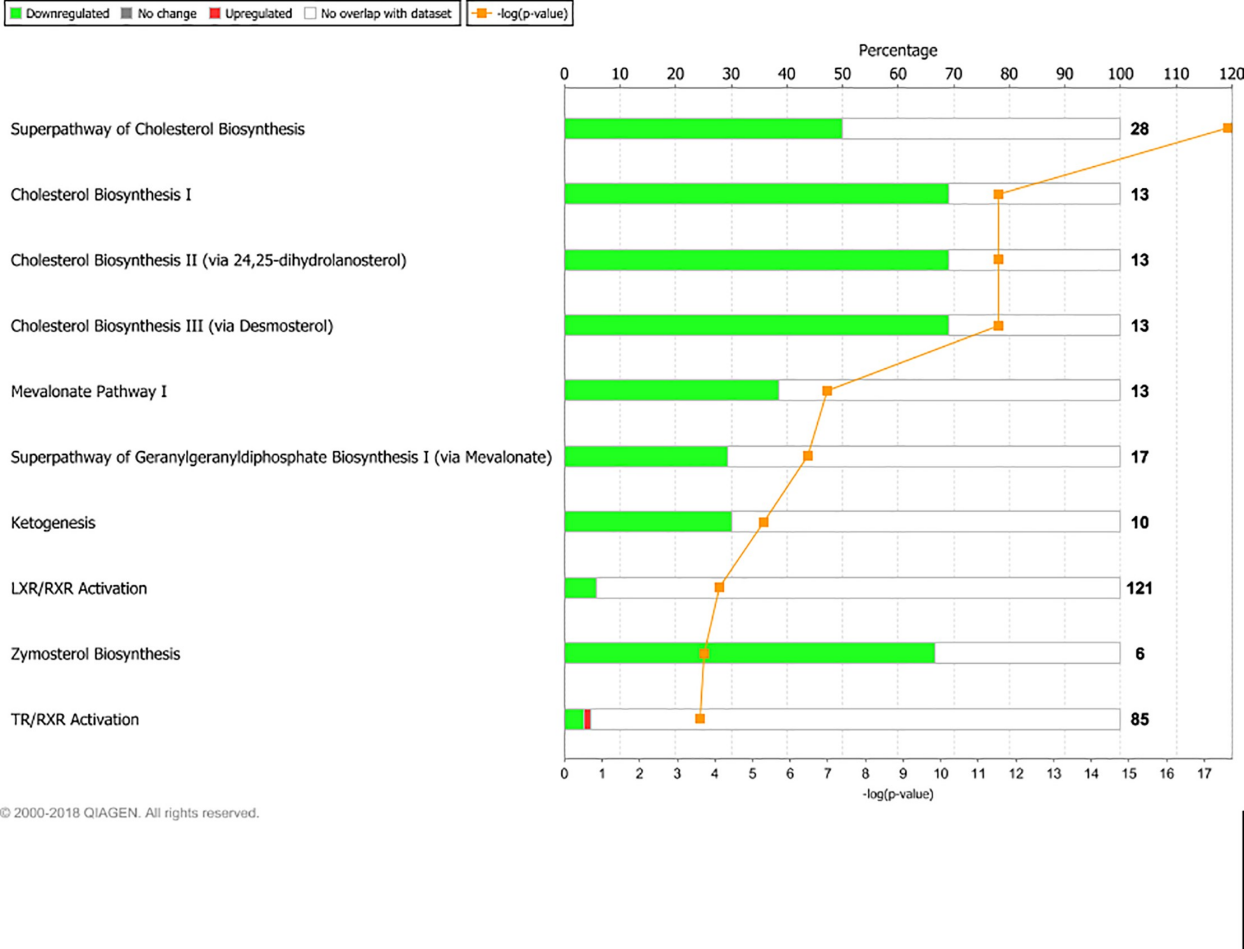
Main pathways affected by differently expressed genes between Low-Protein, High-Energy and Low-Protein, Low-Energy treated heifers at month seven of pregnancy. Main affected pathways between heifers fed Low-Protein, High-Energy (LPHE) or Low-Protein, Low-Energy (LPLE) diets until pregnancy when comparing them at month 7 of gestation. Red bars indicate percent upregulated, and green bars indicate percent downregulated genes pertaining to the pathway in LPHE versus LPLE heifers. Number to the right of bars display total number of genes pertaining to each pathway. Orange squares indicate the negative logarithm of p-value of observation (-log p-value = 1.3 equals p-value = 0.05). (Reprinted from IPA under a CC BY license, with permission from Qiagen Bioinformatics, original copyright 2018).

**Fig 2:**
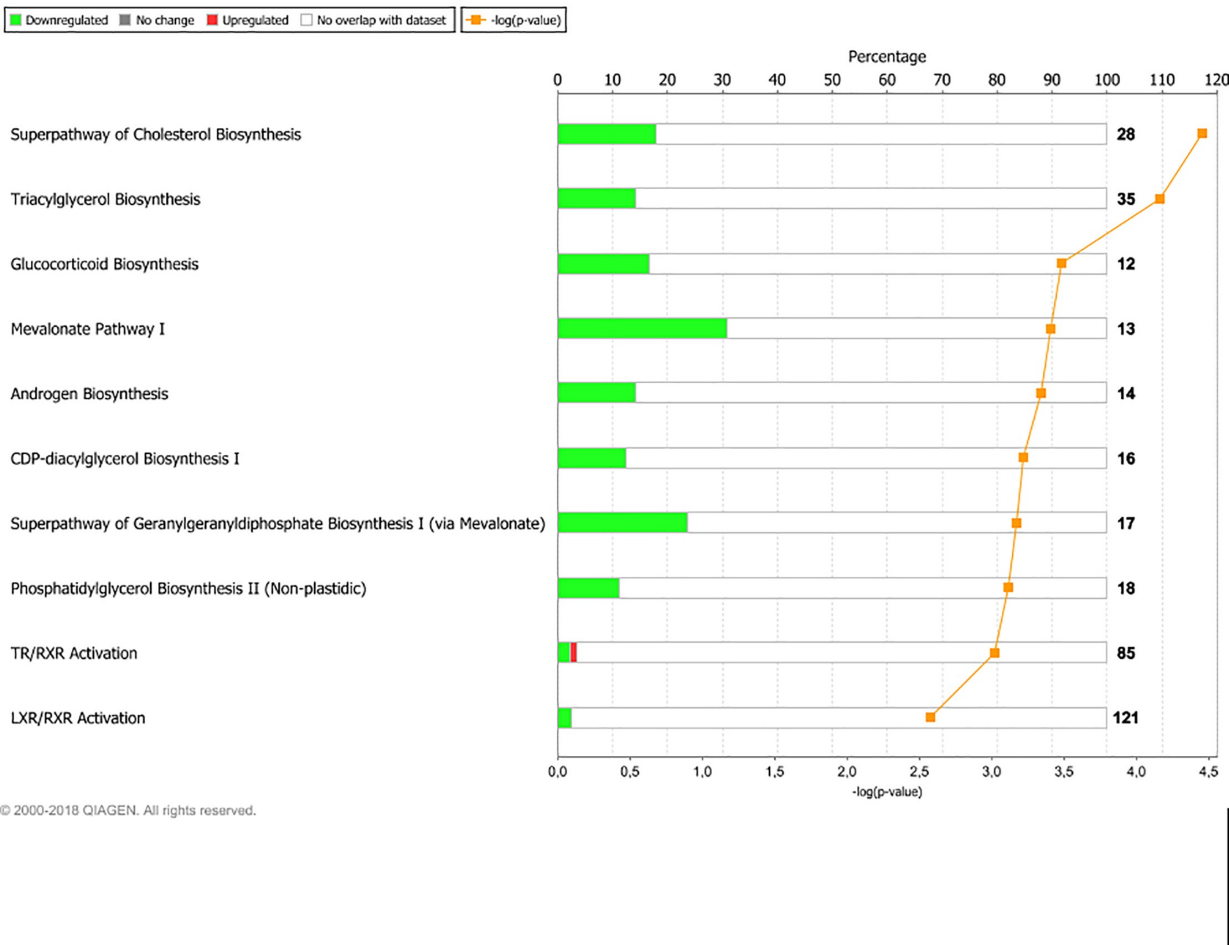
Main pathways affected by differently expressed genes between High-Protein, High-Energy- and High-Protein, Low- Energy heifers at month seven of pregnancy. Main affected pathways between heifers fed High-Protein, High-Energy (HPHE) or High-Protein, Low-Energy (HPLE) diets until pregnancy when comparing them at month 7 of gestation. Red bars indicate percent upregulated, and green bars indicate percent downregulated genes pertaining to the pathway in HPHE versus HPLE heifers. Number to the right of bars display total number of genes pertaining to each pathway. Orange squares indicate the negative logarithm of p-value of observation (-log p-value = 1.3 equals p-value = 0.05). (Reprinted from IPA under a CC BY license, with permission from Qiagen Bioinformatics, original copyright 2018).

Sequencing yielded on average 57.2 million raw reads per sample. Alignment gave an average overall read mapping rate of 95.1% and a concordant pair alignment rate of 89.6%. Genes with a minimum CPM (counts per million) of two in at least six (n = 6) samples were considered to be expressed and were included in the differential expression analysis. Out of 26,740 gene transcripts in the UMD3.1 bovine genome assembly, 13,396 gene transcripts fulfilled this criterion. The DEG lists resulting from the examined contrasts varied greatly in size. The number of DEG found for each contrast is presented in Table 3. At PREG, the LPHE-LPLE and HPHE-HPLE comparisons resulted in 79 and 57 DEG, respectively. The main affected pathways for these comparisons are shown in Figs 1 and 2. The HPLE-LPLE comparison yielded only five DEG, while we found no DEG when comparing HPHE and LPHE. All post-treatment comparison DEG are listed in S1 Table, and complete lists of significantly affected pathways for the LPHE-LPLE and HPHE-HPLE comparisons are given in S2 Table. The HP-LP comparisons yielded no significantly affected pathways.

**Table 3:** Number of differentially expressed genes (DEG) for all examined contrasts.

		DEG	Upregulated	Downregulated	Uncharacterized
Within-treatment changes from 12MO to PREG:					
	12MO-PREG LPHE	3988	2873	1115	226
	12MO-PREG HPHE	3997	2711	1286	244
	12MO-PREG LPLE	534	471	63	31
	12MO-PREG HPLE	334	160	174	19
Between-treatment differences at PREG:					
	LPHE-LPLE	79	21	58	12
	HPHE-HPLE	57	19	38	13
	HPHE-LPHE	0	0	0	0
	HPLE-LPLE	5	0	5	1

DEG numbers for all contrasts analyzed in EdgeR. LPHE = Low-protein, High-energy; HPHE = High-protein, High-energy; LPLE = Low-protein, Low-energy and HPLE = High-protein, Low-energy (HPLE). Upregulated means refers to upregulated in the first element of the contrast relative to the second. Genes with an FDR < 0.05 are defined as DE.

The HE treated heifers underwent a larger change in ration characteristics after conception than heifers on LE treatments and this seems to be reflected in their degree of alteration in gene expression between 12MO and PREG, as these groups displayed more than 7 times as many DEG as any of the LE groups. The 12MO-to-PREG change in gene expression was massive for both HE treatments; The LPHE heifers displayed 3987 DEG, and the HPHE heifers displayed 3997 DEG with an FDR < 0.05. The 12MO-to-PREG gene expression change for the two LE treatments was less pronounced but still considerable, with 534 and 334 DEG (FDR < 0.05) for the LPLE and HPLE groups, respectively. The within-treatment 12MO-to-PREG gene expression changes were larger than the differences found between the dietary treatment groups during 12MO [34], and much larger than any differences found between treatments at PREG. The full lists of DEG between 12MO and PREG are shown in S3–S6 Tables for the LPHE, HPHE, LPLE and HPLE treatments, respectively.

**Fig 3:**
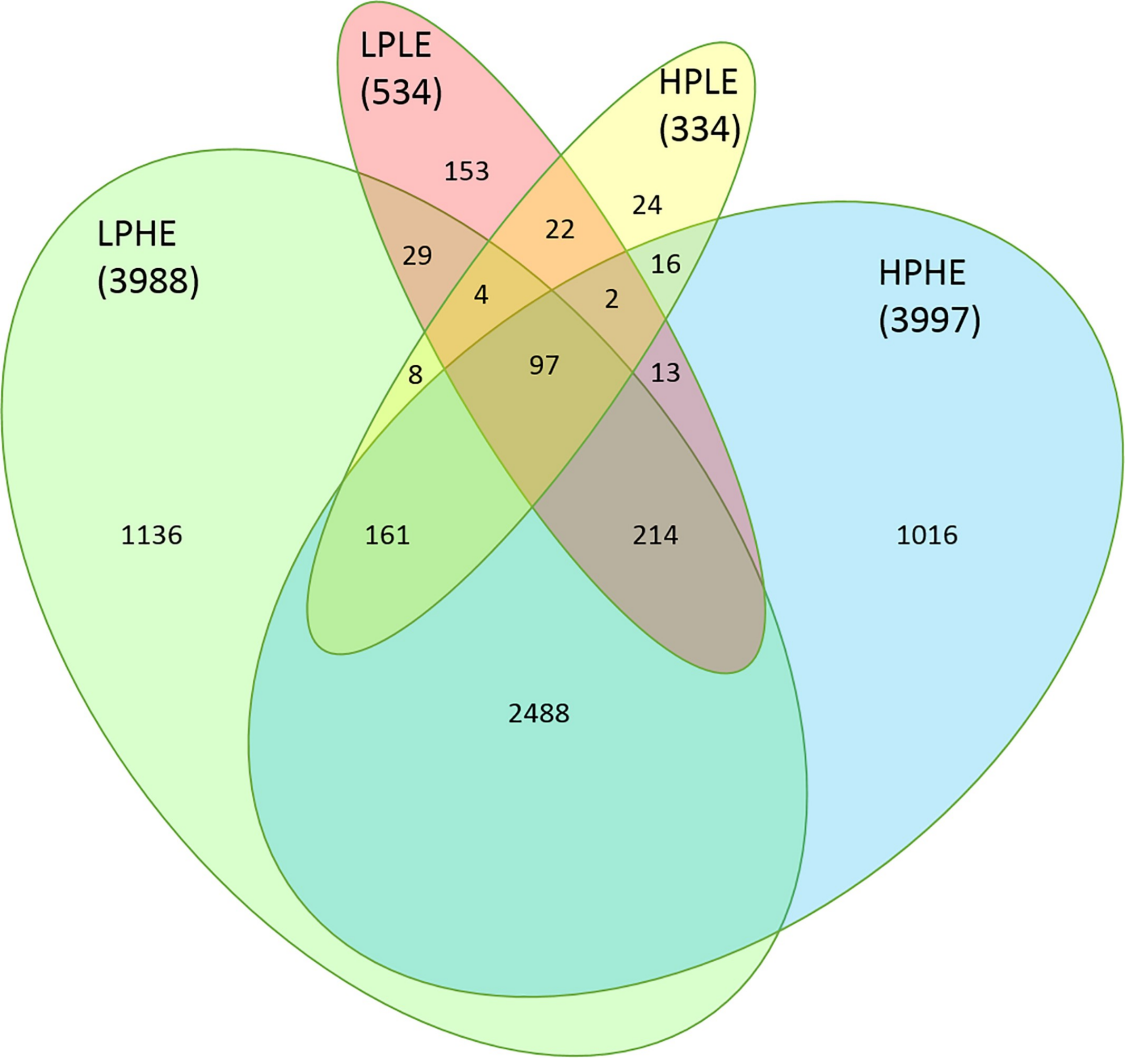
Unique and shared differentially expressed genes (DEG) from the experimental feeding period (12MO) to pregnancy (PREG). The diagram shows the number of differentially expressed genes unique to or shared between experimental treatment groups from experimental feeding at 12 months of age, to month 7 of gestation (at which point all heifers had received the same diet for 6 months). LPHE = Low-protein, High-energy; HPHE = High-protein, High-energy; LPLE = Low-protein, Low-energy and HPLE = High-protein, Low-energy (HPLE). Numbers in parentheses are the total number of differentially expressed genes found for each treatment. (Venn diagram modified from VennDiagram and Venneuler R packages).

**Fig 4:**
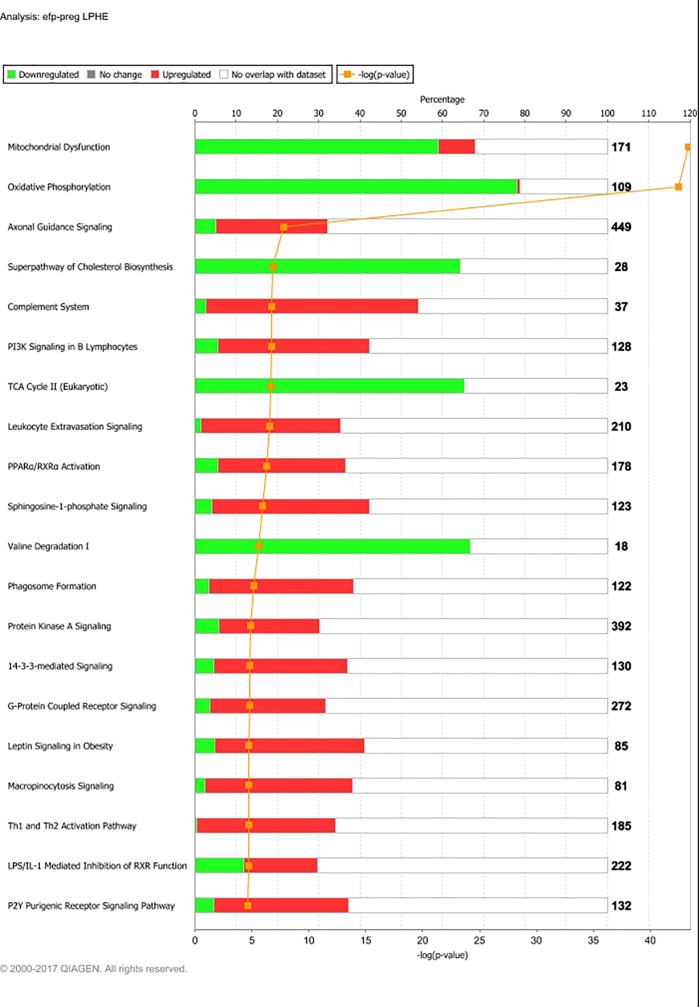
Main affected pathways from experimental feeding period (12MO) to pregnancy (PREG) for Low-protein, High-energy fed heifers (LPHE). Main affected pathways for LPHE heifers during development from experimental feeding at 12 months of age month 7 of gestation (at which point all heifers had received the same diet for 6 months). Red bars indicate percent upregulated, and green bars indicate percent downregulated genes pertaining to the pathway during PREG versus 12MO. Number to the right of bars display total number of genes pertaining to each pathway. Orange squares indicate the negative logarithm of p-value of observation (-log p-value = 1.3 equals p-value = 0.05). (Reprinted from IPA under a CC BY license, with permission from Qiagen Bioinformatics, original copyright 2018).

**Fig 5:**
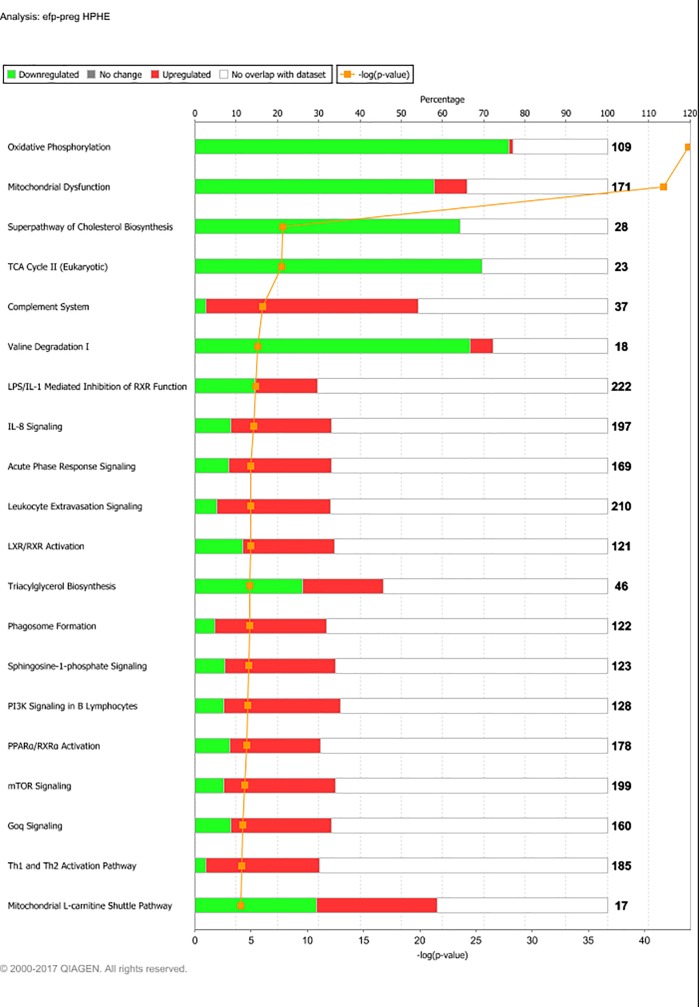
Main affected pathways from experimental feeding period (12MO) to pregnancy (PREG) for High-protein, High-energy fed heifers (HPHE). Main affected pathways for HPHE heifers during development from experimental feeding at 12 months of age, to month 7 of gestation (at which point all heifers had received the same diet for 6 months). Red bars indicate percent upregulated, and green bars indicate percent downregulated genes pertaining to the pathway during PREG versus 12MO. Number to the right of bars display total number of genes pertaining to each pathway. Orange squares indicate the negative logarithm of p-value of observation (-log p-value = 1.3 equals p-value = 0.05). (Reprinted from IPA under a CC BY license, with permission from Qiagen Bioinformatics, original copyright 2018).

**Fig 6:**
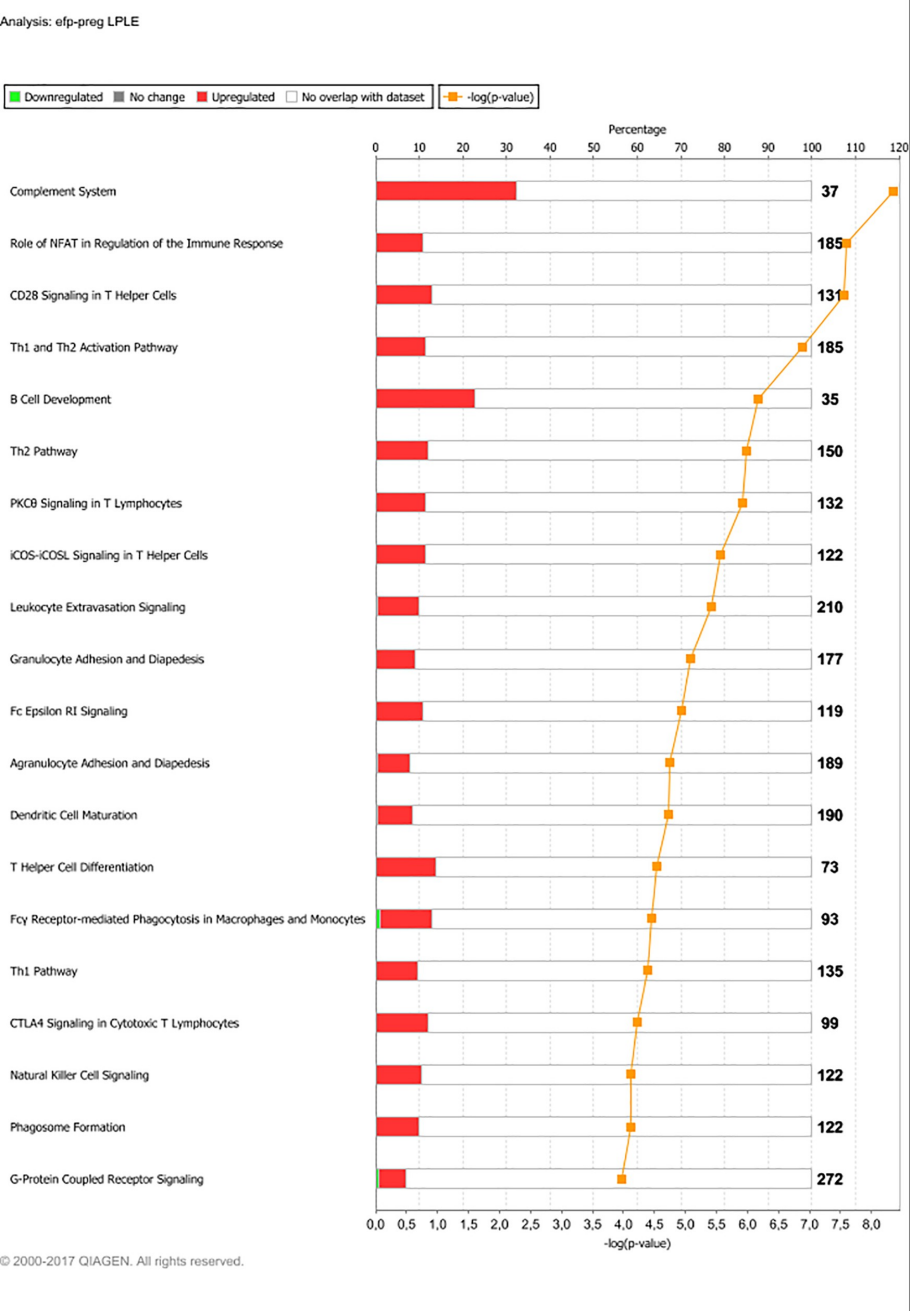
Main affected pathways from experimental feeding period (12MO) to pregnancy (PREG) for Low-protein, Low-energy fed heifers (LPLE). Main affected pathways for LPLE heifers during development from experimental feeding at 12 months of age, to month 7 of gestation (at which point all heifers had received the same diet for 6 months). Red bars indicate percent upregulated, and green bars indicate percent downregulated genes pertaining to the pathway during PREG versus 12MO. Number to the right of bars display total number of genes pertaining to each pathway. Orange squares indicate the negative logarithm of p-value of observation (-log p-value = 1.3 equals p-value = 0.05). (Reprinted from IPA under a CC BY license, with permission from Qiagen Bioinformatics, original copyright 2018).

**Fig 7:**
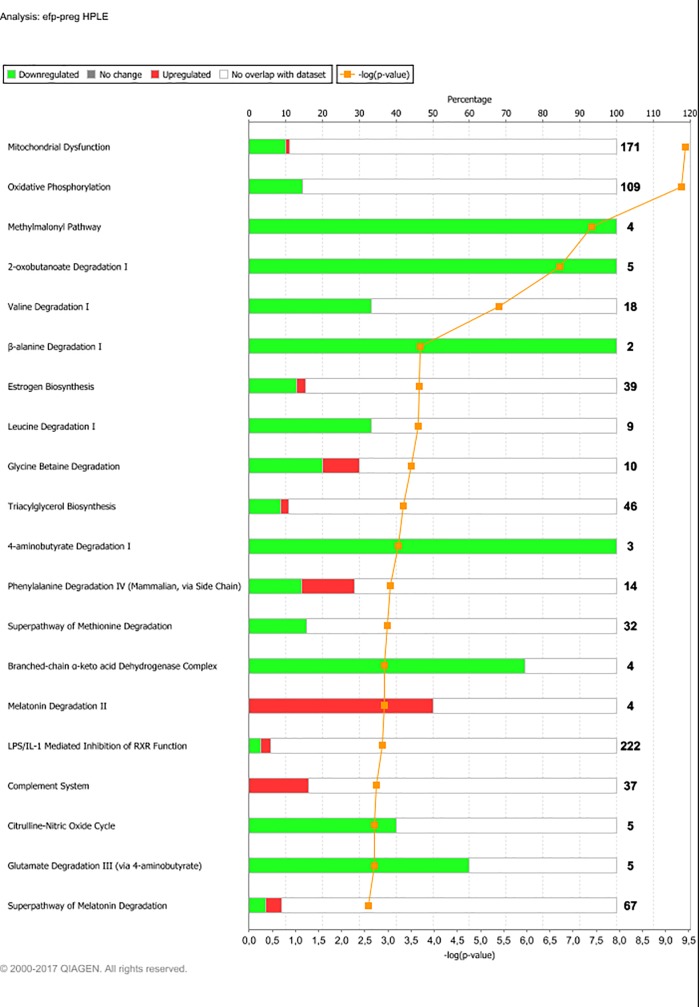
Main affected pathways from experimental feeding period (12MO) to pregnancy (PREG) for High-protein, Low-energy fed heifers (HPLE). Main affected pathways for HPLE heifers during development from experimental feeding at 12 months of age, to month 7 of gestation (at which point all heifers had received the same diet for 6 months). Red bars indicate percent upregulated, and green bars indicate percent downregulated genes pertaining to the pathway during PREG versus 12MO. Number to the right of bars display total number of genes pertaining to each pathway. Orange squares indicate the negative logarithm of p-value of observation (-log p-value = 1.3 equals p-value = 0.05). (Reprinted from IPA under a CC BY license, with permission from Qiagen Bioinformatics, original copyright 2018).

As illustrated in Fig 3, the 12MO-to-PREG DEG for each group displayed a complex pattern of overlapping: The two HE groups had the greatest change in total gene expression. Out of the 5184 DEG found for these two groups in total, 66% of the DEG were common to both groups. The two LE groups displayed a much smaller overall gene expression change, but comparable fractions of shared and unique DEG. IPA pathway analysis yielded 506, 493, 314 and 215 significantly affected pathways for LPHE, HPHE, LPLE and HPLE heifers from 12MO to PREG, respectively. Complete lists of significantly affected pathways for each treatment group are presented in S7–S10 Tables. The main over-represented pathways for each treatment group from 12MO to PREG are shown in Figs 4–7.

### Post-treatment differences between dietary treatments

Although offered the same feed ad libitum during pregnancy and entering pregnancy at a similar BW, the HE and LE heifers were not the same age (they were inseminated at about 13 and 17 months of age), they did not respond to the pregnancy diet in the same way (LE heifers grew faster than HE heifers) and consequently seemed to be in two different physiological states during pregnancy. The LE-treated heifers maintained the same growth in response to the new ad libitum pregnancy feeding compared to the restricted rations they were offered earlier. As BCS also remained constant for the LE heifers through pregnancy, their growth may be assumed to consist of proportional amounts of lean and fat tissue (Table 2), but our study does not allow for precise assessment of lean and fat tissue distribution. Compared to the experimental feeding period, the HE-treated heifers decreased their growth rate and BCS during pregnancy. The LPHE-LPLE and HPHE-HPLE comparisons resulted in 79 and 57 DEG, respectively. Cholesterol biosynthesis, thyroid receptor complex (TR/RXR) activation, liver X receptor (LXR/RXR) activation and sirtuin signaling pathways were among the pathways which were significantly affected in both comparisons, with the majority of constituent genes displaying decreased expression in the HE- compared to the LE-heifers. Sirtuins are a class of proteins involved in a wide range of cellular processes, including ageing, transcription, apoptosis, inflammation, energy efficiency and adaptation to low calorie intake [42–44]. The TR/RXR and LXR/RXR activation pathways are involved in the regulation of both lipid, carbohydrate and steroid metabolism [45, 46], and the latter (cholesterol metabolism) was significantly affected and downregulated in HE heifers in both comparisons. As the HE heifers decreased their BCS through pregnancy, a decreased need for cholesterol in adipose cell membranes and downregulation of its production is logical.

The TR/RXR and LXR/RXR activation and sirtuin signaling pathways were represented mainly by genes which are also highly involved in energy metabolism pathways, such as *ACACA*, *FASN*, *ACLY*, *ACSS2*, *G6PD* and *SCD*, and no genes coding directly for sirtuins, thyroid or liver X receptors or RXR complexes were DE. Therefore, we cannot ascertain whether the downregulation of these genes in HE heifers is mainly associated with cell signaling or with metabolic pathways.

A gene coding for an uncoupling protein, *UCP2*, is also associated with TR/RXR, LXR/RXR and Sirtuin signaling pathways and was upregulated in HE heifers in both comparisons. Uncoupling proteins are embedded in the inner mitochondrial wall of many cell types, but has been most extensively described in brown adipose tissue, where UCP1 is a prerequisite for the essential heat production function of this tissue [47]. The uncoupling proteins function as proton carriers mediating a regulated proton leakage through the inner mitochondrial membrane, free-coupled from the reactions of the respiratory chain [48]. Free-coupling proton flow from ATP production in the respiratory chain causes a futile cycle where energy spent on pumping protons against an electrical gradient is lost as the protons flow back out along the gradient without energy-rich molecules being produced. However, the roles of the other UCPs (UCP2-5) seem to be more complex than the well-known thermogenetic function of UCP1 [49]. UCP2 has been found to act as a free radical scavenger, and it also plays a role in regulating insulin [49].

The *UCP2* gene is almost ubiquitously expressed throughout body tissues [49]. It is regulated by TR and PPARγ and was recently shown to be regulated by diet in subcutaneous white adipose tissue of cattle [50]. However, our heifers had been offered the same diet for 6 mo before PREG biopsises were taken. Thus, the observed upregulation of *UCP2* in HE heifers is not a direct, contemporary effect of diet, and the cause of this post-treatment effect is unclear. It may represent a lasting change in the regulation of insulin secretion and glucose metabolism or an increased need for free radical scavenging because of a higher lipid turnover (BCS decrease) in the adipose tissue of pregnant HE heifers, but further studies are required to confirm or reject these hypotheses.

The difference in gene expression between HPLE and LPLE heifers, although small, increased post-treatment; from zero at 12MO to five at PREG. This is surprising but is in agreement with the results of Van Amburgh et al, who found that post-treatment effects of prepubertal feeding regimes differing in energy and protein content had a larger impact on subsequent milk yield than the experimental feeding itself [32].

Three DEG maintained their direction of differential expression between treatment groups from 12MO to pregnancy, despite the feed change, the age difference and the different growth profiles obtained during pregnancy. Two of these genes were found to be DEG in the LPHE-LPLE comparison. The first gene was *RNS1*, coding for pancreatic ribonuclease. This was upregulated in LPHE relative to LPLE heifers at 12MO as well as PREG with an FDR<0.02, and fold changes of 2.5 and 5.7 at 12MO and PREG, respectively. The other gene found for the LPHE-LPLE contrasts was *ENSBTAG00000007075*, coding for a major histocompatibility complex class I (A-like precursor). This gene was also upregulated in LPHE heifers relative to LPLE heifers at both time points with an FDR<0.02. Log 2 fold change was 3.4 at 12MO and 12.0 at PREG. The third gene displaying a persisting treatment effect at PREG was *ENSBTAG00000048049*, a novel gene with immunoglobulin-like sequences, with several orthologues in other species found to be an *IGHM* gene [51]. This was downregulated for HPHE with a log 2 fold change of -2.9 and -6.1 in the HPHE-HPLE contrast at 12MO and PREG, respectively, and with an FDR <0.02 at both time points. As bovine pancreatic ribonuclease represents a very basic and general cellular function, it is hard to deduce exactly what function a persisting expression difference represents in the adipose tissue of heifers in our study. Furthermore, this gene was also one of the genes commonly upregulated across all treatment groups from 12MO to PREG. This indicates that several mechanisms may have influenced the expression of this gene, and the apparently persistent treatment effect may in fact be the result of different underlying causes. The two immunologically related genes on the other hand displayed different, treatment-dependent changes from 12MO to PREG as well as the persistent DE between treatment groups at these time points. Together with the large expression differences (log 2 fold change), this may indicate that the experimental diets have in fact elicited a lasting difference in the expression of *ENSBTAG00000007075* and *ENSBTAG00000048049*. This may suggest an ongoing potential for some type of epigenetic programming acting through an interaction between nutrition and the immune system after three months of age, which is when the experimental treatments were initiated. To our knowledge, this has not been shown before, but further studies are required to either confirm or reject this hypothesis.

### Within-treatment gene expression changes from 12MO to PREG

#### LPHE and HPHE treatments

For the LPHE and HPHE heifers, the gene expression changes occurring between 12MO and PREG were very similar. Apart from slight differences in the ranking of the significantly affected pathways and number of DEG, the main pathway results were nearly identical. Therefore, gene expression changes for these two treatments are presented and discussed commonly in this section, under the term HE treatments (Any specific changes pertaining only to one group are specified).

Gene expression changes from 12MO to PREG for the HE treatments were dominated by a downregulation of pathways pertaining to energy metabolism and mitochondrial functions, namely oxidative phosphorylation, mitochondrial dysfunction, cholesterol biosynthesis and the TCA cycle. Fifteen genes representing all eight steps in the TCA cycle and 85 genes associated with either of the five complexes in the respiratory chain were significantly downregulated. This finding is in concordance with our observations from the LPHE-LPLE contrast at 12MO: At this point, oxidative phosphorylation, mitochondrial dysfunction and the TCA cycle was clearly upregulated in the high-energy fed LPHE heifers compared to the restrictively fed LPLE heifers [34]. The transition to the pregnancy diet implied a reduction in energy intake for the HE heifers. This possibly explains a downregulation of pathways connected to the production of energy-rich molecules available for lipid synthesis.

A small proportion of the DEG associated with mitochondrial dysfunction were upregulated instead of downregulated. None of these genes were directly associated with energy-yielding reactions, and therefore do not change the overall massive downregulation of genes associated with energy production in the adipose of HE heifers. However, among the few upregulated DEG pertaining to mitochondrial dysfunction were *NCSTN*, *APHA1*, *BACE1*, *APP*, *MAOB* and *CASP9*. These genes are all part of a mitochondrial pathway leading to production of amyloid-beta from the amyloid precursor protein (APP) and apoptosis [52]. Such changes are associated with oxidative stress and lipid peroxidation [53], but studies on this pathway has so far been performed mainly with a focus on human diseases and little is known about its function in the adipose tissue of ruminants. However, apoptosis induced by caspases have also been associated with programmed cell death in lipoatrophy [54]. These DEG may therefore be an indication of apoptosis associated with the decreasing fatness of the HE heifers from 12MO to PREG (Table 2).

Cholesterol biosynthesis was a major affected pathway for the HE treatments. 17 out of 28 genes pertaining to this pathway were downregulated at PREG in HE treatments. Cholesterol is synthesized in adipose tissue, where it is stored as a free sterol in the phospholipid membrane of lipid droplets [55]. Thus, it is logical to find a downregulation of cholesterol synthesizing pathways in adipose tissue when dietary energy is reduced.

Besides metabolic pathways, the list of highly affected pathways for the HE treatments was dominated by signaling pathways, and the one most significantly affected for the LPHE heifers was axonal guidance signaling (144 DEG). PI3K signaling in B lymphocytes (55 DEG) and leukocyte extravasation signaling (71 DEG) was highly ranked for both HE treatments.

The axonal guidance pathway was represented by 121 upregulated and 23 downregulated DEG for the LPHE heifers. Axonal guidance refers to the directional guidance of axons towards their target tissues or cells during tissue development or restructuring. White adipose tissue is sympathetically innervated, and the sympathetic system has been shown to regulate lipolysis [56]. Axonal guidance is signalled via a large range of molecules, collectively named “guidance cues” which includes semaphorins, netrins, ephrins, SLIT ligands, morphogens (Wnt), growth factors, EPH receptors, ROBO receptors and cytokines [57, 58]. Genes coding for all these types of molecules were upregulated in HE heifers at PREG. Even though there are no neuronal cell bodies present in adipose tissue, the mRNA reads for these gene products in our data may originate from neurons, as transport to and local translation of neuronal mRNA in axons, far away from the nuclei in which they were transcribed, constitutes an important regulation mechanism in axon guidance [58]. As the axonal guidance pathway was significantly upregulated in LPHE heifers, in contrast to all other treatment groups, we assume it to be affected by their dietary treatment. If so, it is reasonable to assume that axonal guidance occurred to a greater degree in this group because these heifers experienced the greatest alteration in diet and the largest decrease in BCS causing these heifers to have the greatest degree of adipose tissue restructuring.

Phosphatidylinositol 3-kinases (PI3K) exert their effects on membrane signaling complexes within many pathways and systems [59, 60]. In B lymphocytes, PI3K regulates both activation, proliferation and differentiation [59]. More recently, PI3K signaling has also been shown to play an important role in energy balance regulation: Experimentally induced reduction of PI3K signaling increases energy expenditure and protects from obesity and obesity-related disorders [60, 61]. Thus, the upregulation of PI3K signaling in adipose tissue of HE heifers after transition to low-energy feeding may be part of an energy-saving strategy. If so, manipulation of PI3K signalling may provide a means for increased feed efficiency in cattle, the opposite goal of ongoing human research, which focuses on alleviating obesity by increasing energy expenditure through PI3K functions.

Leukocyte extravasation and immune cell infiltration of adipose tissue has been the subject of intense research over the last decades, as obesity, metabolic syndrome and adipose tissue dysfunction in humans is associated with low-grade chronic inflammation and increased immune cell trafficking of adipose tissue [62]. In contrast, our results show an upregulation of leukocyte extravasation signals in animals subjected to a reduction in feed energy, whilst displaying no increase in adiposity. This is in line with recent research on both cattle and other species, stating that adipocyte inflammation is not only associated with adipocyte-related diseases, but is also an essential part of physiological tissue remodeling, especially during lipolysis [63–67]. The type, degree, and duration of leukocyte migration and infiltration is interdependent on the type of physiological or pathological change occurring in the adipose tissue [62, 63]. Current knowledge indicate that in dairy cattle, differences in immune cell infiltration in adipose tissue seem to be affected by stage of lactation [65–67], body condition score [68, 69] and the presence, degree and underlying cause (dietary, physiological or pathological) of a positive or negative energy balance [64, 66, 67, 69, 70].

#### LPLE

The LPLE treatment expression changes from 12MO to PREG clearly differed from the other three dietary treatments: The LPLE experimental diet was the one most similar to the pregnancy diet, and this seems to be reflected in the gene expression of the LPLE heifers through the lack of affected metabolic pathways. The top list of affected pathways due to/or during diet change for this treatment group almost exclusively consisted of pathways associated with immunological and inflammatory functions (Fig 6). It is also worth mentioning that out of the eleven most affected pathways for this group, six were specifically associated with T lymphocytes (CD28 signaling in T helper cells, NFAT regulation of the immune response, PKCθ signaling in T-lymphocytes, iCOS-iCOSL signaling in T helper cells, Th1 and Th2 activation and Th2 pathway). The type of immune cells involved in adipose tissue infiltration is dependent on the current stage and physiology / pathology of adipose tissue [62]. T-cells infiltrating the adipose tissue has been described as a primary event in the process of macrophage recruitment, adipose inflammation and insulin resistance [71, 72]. However, the LPLE heifers were never obese nor clinically ill during our trial. The T-cell marker CD3 is also found in both omental and subcutaneous adipose tissue of dairy cows. However, the occurrence was higher in the omental adipose tissue of dairy cows suffering from abomasal displacement. Together with our findings, this may indicate that T-cell infiltration in bovine adipose tissue is both a part of normal, physiological processes such as adipose tissue remodeling, as well as pathological inflammatory conditions [64, 72].

#### HPLE

For the HPLE heifers, the diet change from their experimental feed treatment to the pregnancy feed meant a transition to a ration of lower protein density. From 12MO to PREG, we observed a downregulation of several pathways associated with amino acid degradation in the HPLE heifers; As shown in Fig 7, 11 of the 20 most highly affected pathways were implicated in the degradation of valine (Val), leucine (Leu), methionine (Met), alanine (Ala), phenylalanine (Phe), glycine (Gly) and glutamate (Glu). Except for Met and Gly, showing a mixed pattern of up- and downregulated DEG, all DEG pertaining to these pathways were downregulated at PREG. No other treatments displayed a similar downregulation of amino acid degrading pathways at PREG, and the reason for HPLE heifers to do so remains unknown. However, we may propose several hypotheses: The capacity of adipose tissue to both synthesize and degrade AA in reaction to concentrations of insulin, glucose or specific AA as part of whole-body metabolism and regulation has been known for decades [73–76]. The pregnancy diet and the experimental diet given to the HPLE heifers differed both in protein content and protein sources (because of the inclusion of concentrate in the experimental diet). Thus, both the total and relative amounts of different AA available to the animals could be expected to differ between the two diets, and the allocation and metabolic fate of specific AA likewise. However, most of the AA represented by significantly affected degradation pathways also have important functions other than acting merely as protein precursors and energy substrates: Methionine also acts as a methyl donor for transmethylation reactions in lipid biosynthesis and this function may also be the cause of Met degradation in the adipose tissue of HPLE heifers [77]. Levels of circulating branched chain amino acids (BCAA; Val, Leu, Isoleu) are associated with metabolic regulation, obesity and insulin resistance [78, 79], and are regulated by BCAA metabolism in adipose tissue [80]. The other AAs represented by downregulated degradation pathways (Phe, Ala, Gly and Glu) also possess vital functions in addition to being energy substrates and protein precursors [81], and the background for the downregulation of their degradation pathways at PREG is almost certainly more complex than a simple up- and downregulation because of their availability as protein precursors in peripheral tissues. However, as HPLE was the only group displaying this effect, it must be assumed to be dietary related.

The HPLE heifers received a diet of similar energy content but more protein than the LPLE heifers at 12MO, and the net energy intake for HPLE was very similar to that of the LPLE heifers (Table 1). Therefore, one would expect the change in energy intake from 12MO to PREG for the two LE groups to be similar, and small. Interestingly, the HPLE heifers still displayed a pathway analysis result with a closer resemblance to the results for the HE treatments, although at a smaller scale, with a downregulation of mitochondrial dysfunction and oxidative phosphorylation indicating a decrease in energy flux towards adipose tissue. Together with the downregulation of amino acid degradation pathways this indicates that the effect of total ration characteristics during 12MO has a larger effect than dietary energy and protein in isolation.

#### Changes from 12MO to PREG common to all groups

Among the genes found to be differentially expressed between 12MO and PREG samples for all four groups, 97 displayed a similar up- or down-regulation in all groups. These genes are listed in S11 Table. Despite different feed treatments until confirmed pregnancy, different growth rates both before and during pregnancy, and different age at PREG, all heifers also had some factors in common over this period; all heifers became older, they all grew (though at different rates), they all became pregnant and at PREG they had all been offered the same feed *ad lib* for about 6 months.

**Fig 8:**
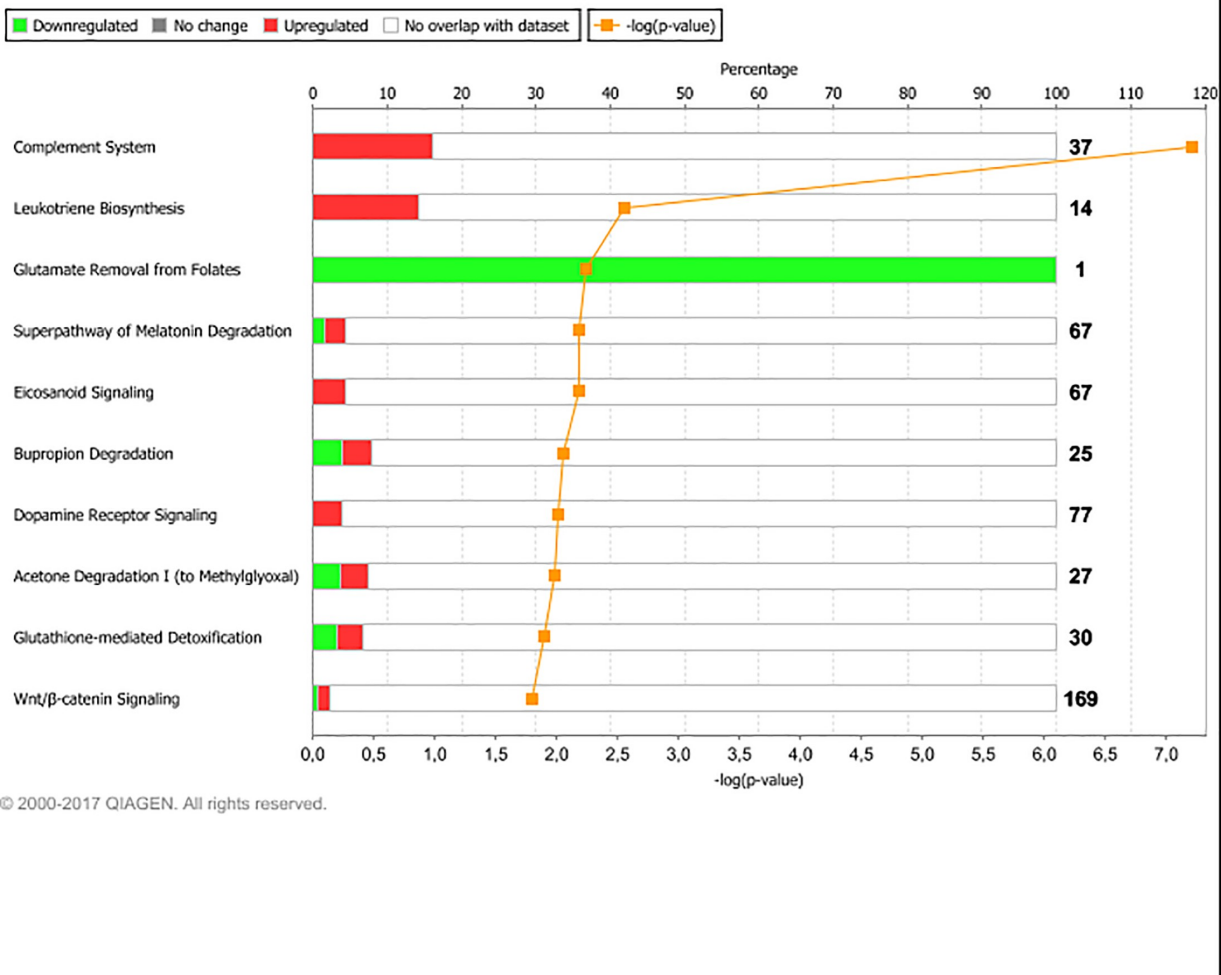
Main affected pathways common to all treatments during the period from 12MO to PREG. Main affected pathways associated with differentially expressed genes common to all heifers during development from experimental feeding at 12 months of age, to established similar feeding regimes at month 7 of gestation. Red bars indicate percent upregulated, and green bars indicate percent downregulated genes pertaining to the pathway during PREG versus 12MO. Number to the right of bars display total number of genes pertaining to each pathway. Orange squares indicate the negative logarithm of p-value of observation (-log p-value = 1.3 equals p-value = 0.05). (Reprinted from IPA under a CC BY license, with permission from Qiagen Bioinformatics, original copyright 2018).

We interpret the differential expression of these common genes to be treatment-independent, and that they were caused by pregnancy, general progression of age and/or growth or by characteristics of the pregnancy feed per se. Pathway analysis of the common DEG yielded 23 significantly over-represented pathways. The main pathways are shown in Fig 8, and among these were the complement system, leucotriene biosynthesis and eicosanoid signaling. The immune system is known to undergo several changes during pregnancy, in order to avoid rejection and expulsion of the fetus while maintaining an adequate immune competence to combat infections and other threats. [82–85]. A characteristic part of these changes seems to be an activation of the innate immune system, including the complement system and natural killer cells [83, 84, 86]. Adipsin (also known as Complement factor D, coded by *CFD*, upregulated in PREG heifers), has been subject to extra interest as it has been associated with pre-eclampsia and abortion [87–89]. Consequently it seems complement activation may be part of both physiological and pathological immune changes related to pregnancy.

The upregulation of genes coding for arachidonate 5-lipoxygenase (*ALOX5*) and its activating protein (*ALOX5AP*) together with leukotriene C4 synthase (*LTC4*) indicates an increased leukotriene production, especially of LTC4, from arachidonic acid in adipose tissue during pregnancy. Expression changes of *ALOX5* and *ALOX5AP* through gestation and during labour in human chorionic membranes have been shown, suggesting a role for these genes both at parturition as well as earlier in pregnancy [90]. Concentration of LTC4 has also been shown to increase in human amniotic fluid during labour [91]. An increased capacity for leukotriene synthesis during pregnancy has recently been detected in human blood [82], but so far, no studies have reported such effects in adipose tissue. As the heifers in our study underwent normal pregnancies we assume the observed changes in their gene expression to be physiological. However, further studies should be performed to establish whether any of these genes are potential biomarkers for gestational health or preservation.

#### Upstream regulators

**Fig 9:**
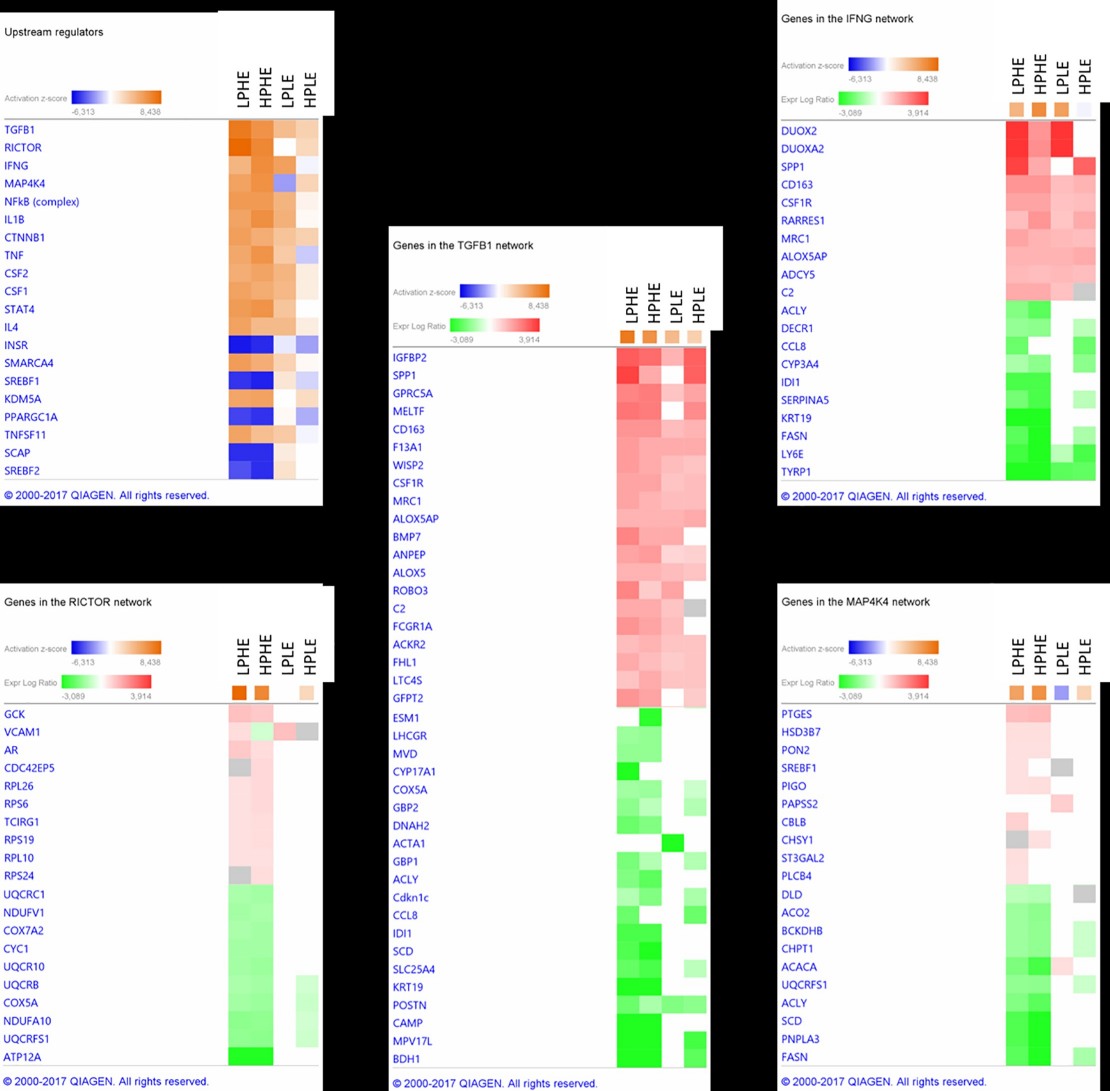
Heatmap displaying top 20 upstream regulators active across all treatments from the experimental feeding period (12MO, 12 mo) to month 7 of pregnancy (PREG, 20 (HE) and 24 (LE) mo) and most highly differentially expressed genes (DEG) within each treatment for the top 4 upstream regulators. LPHE = Low-protein, High-energy; HPHE = High-protein, High-energy; LPLE = Low-protein, Low-energy and HPLE = High-protein, Low-energy (HPLE). LogFC = log 2 Fold Change. a) Heatmap of upstream regulators with activation z-score. Orange squares indicate activation, blue squares indicate deactivation of the regulator functions. A -2 ≥ z-score ≤ 2 indicates that the activation /deactivation was statistically significant (p < 0.05). b) Most highly up- or downregulated genes pertaining to upstream regulator Transforming Growth Factor 1 (TGFB1), as identified by IPA comparison analysis. Red/pink squares indicate upregulation, green squares indicate downregulation as measured by logFC. c) Most highly up- or downregulated genes pertaining to the upstream regulator Rapamycin-insensitive companion of mammalian target of rapamycin (RICTOR), as identified by IPA comparison analysis. Red/pink squares indicate upregulation, green squares indicate downregulation as measured by logFC. d) Most highly up- or downregulated genes pertaining to upstream regulator interferon gamma (IFNG) as identified by IPA comparison analysis. Red/pink squares indicate upregulation, green squares indicate downregulation as measured by logFC. e) Most highly up- or downregulated genes pertaining to the upstream regulator mitogen-acticated protein kinase 4 (MAP4K4), as identified by IPA comparison analysis. Red/pink squares indicate upregulation, green squares indicate downregulation as measured by logFC. (Modified from IPA under a CC BY license, with permission from Qiagen Bioinformatics, original copyright 2018).

Despite the differences in DEG, growth rates, age and BCS development during the period from 12MO to PREG, the treatment groups displayed many similarities in their main upstream regulators as analyzed by IPA. A heatmap containing key regulators and their most highly affected DEG is shown in Fig 9. The comparison analysis demonstrated transforming growth factor-β 1 (TGFB1) to be the highest ranked upstream regulator of identified DEG overall. At PREG versus 12MO, the *TGFB1* gene itself was significantly upregulated in the adipose tissue of the HE groups, but not in the LE groups. In the HE groups, TGFB1 was directly or indirectly associated with > 450 of the identified DEG between 12MO and PREG. These results indicate a widespread involvement in several cell functions. In adipose tissue, TGFB1 is mainly secreted by cells other than adipocytes, and an increased secretion of TGFB1 has been associated with obesity in other species [92]. In our study TGFB1 was upregulated only in the HE heifers at PREG when comparing to 12MO. This may seem paradoxical as the HE heifers underwent a decrease in energy intake and BCS during the period, but still suggest an association between TGFB1 and adiposity. Gene polymorphisms and plasma levels of TGFB1 have also shown associaton with the risk of preeclampsia and recurrent abortions in humans [93]. As such, it would be interesting to investigate whether TGFB1 or any of its affected downstream molecules proves to be indicators of pregnancy preservation capabilities in cattle as it is in humans.

Rapamycin-insensitive companion of mechanistic target of rapamycin (RICTOR), encoded by *RICTOR*, is part of the mTORC2 protein complex. mTORC2 have both functional and structural similarities to the more thoroughly studied mTORC1, which regulates cell growth based on nutritional status and growth factor-derived signals. mTORC2 has so far been linked more tightly to regulatory functions within the insulin / PI3K signaling pathway [94]. Within mTORC2, RICTOR is thought to have a role as a facilitator for substrate binding and correct subcellular location of mTORC2, and is therefore essential for the function of the mTORC2 complex [95] RICTOR seem to be highly involved in the changes occurring in the HE-treated heifers from 12MO to PREG, but much less so in the LE-treated heifers. An IPA core analysis run on the DEG associated with RICTOR in the HE heifers showed it to be mostly involved in downregulation of genes associated with mitochondrial energy production. These functions were downregulated in the HE heifers compared to the LE heifers probably due to the larger reduction in dietary energy for these groups. This may explain the difference in RICTOR involvement between the two energy treatments, and suggest RICTOR and mTORC2 to have an important role in the regulation of energy production in adipose tissue.

Interferon Gamma (IFNG) is a cytokine regulating a wide range of immune and inflammatory functions. In our study, IFNG was most highly associated with function of T cells, the complement system, cell differentiation and maturation and interleukin signaling across treatments. It was least active in HPLE heifers and most in HPHE and LPLE. Compared to the other treatments, the overall impact of IFNG in the HPLE heifers was also slightly skewed with involvement in citrulline metabolism highly ranked together with a more narrow range of immune functions, such as the complement system and phagosome formation. The reason for this is not known.

Mitogen-activated protein kinase 4 (MAP4K4) displayed increased activity from 12MO to PREG for all treatments except LPLE. The activation also seemed lower for HPLE heifers than for the HE treatments. As for RICTOR, this seem to be explained by the DEG associated with MAP4K4 and to the changes occurring within each treatment group from 12MO to PREG: MAP4K4 was mostly associated with DEG pertaining to energy production, and these were downregulated in HE heifers and to a lesser degree in HPLE heifers (Figs 4, 5 and 7). The LPLE heifers, which underwent the smallest dietary change, did not display such changes (Fig 6). Thus, the regulating function of MAP4K4 in our experimental heifers may have been mainly to decrease the production of energy rich compounds in adipose tissue when subjected to a decrease in dietary energy flux.

Tumor necrosis factor (TNF) was also ranked high among the upstream regulators for all groups. Tumor necrosis factors are a family of multifunctional cytokines with known effects on apoptosis, inflammation, lipid metabolism and cellular redox balance among others [96]. In the context of our study, it is of interest that TNF is an activator of leukotriene production pathways, which were upregulated in all groups at PREG (Figs 4–7 and 9). TNF also acts upon TP53, and mediates some of its effects through this transcription factor [97], and TP53 was also ranked very high among upstream regulators for all treatment groups. *TNF* itself was not DE for any group, but all groups displayed between three and 19 DEG associated with activity regulation of TNF such as *TNFAIP3*, *TNFAIP6*, *C1QTNF6*, *TNFSF18*, *LITAF*, *TNIP2*, *TRAF5* and *TNFRSF1B*. This is a good example of the versatility and flexibility of gene regulation: It may act directly upon the gene coding for a main regulator (as in this case with *TGF1B*), or it may act upon several other genes coding for regulators of the regulator (as here with TNF).

### Summary

Gene expression differences between heifers fed different levels of energy and protein are evident even 6 months after the termination of different feeding regimes and transition to the same diet. Only a few of the gene differences observed while being fed different diets are still present post-treatment. These may indicate a possible immunologic or epigenetic programming, while most post-treatment differences seem to be reflections of different physiological responses to the new diet, rather than the dietary content itself. Still, diet also has a major direct impact on adipose gene expression, which is clear from the treatment-dependent and varied gene expression changes occurring within treatment groups from experimental feeding at 12 months of age until similar feeding at 7 months pregnant. Pregnancy is known to cause immunologic changes, and in the adipose tissue of our heifers, these changes seem to consist mainly of complement activation and increased leukotriene synthesis.Through our study, we have identified several candidate genes and key gene regulators which should be studied further with regards to pregnancy preservation (TGFB1, *CFD*) and metabolic regulation and efficiency (*UCP2*, PI3K, RICTOR, MAP4K4,) in dairy cattle. As this is a purely transcriptomic study, it represents a first step towards new knowledge about adipose tissue responses to diet and pregnancy in cattle, and warrants further studies in terms of functional assays, metabolomics and/ or proteomics for phenotypic confirmation.

## Supporting information

S1 Table Post-treatment differences between dietary treatments. All differentially expressed genes (DEG) from energy treatment comparisons at month 7 of pregnancy.
(XLSX)

S2 Table All pathways affected by differently expressed genes between treatment comparisons at month seven of pregnancy. (XLSX)

S3 Table All DEG (FDR<0.05) between experimental feeding period (12MO) and month 7 of pregnancy (PREG) for heifers fed a high-protein, high-energy diet (HPHE) during the experimental feeding period. (XLSX)

S4 Table All DEG (FDR<0.05) between experimental feeding period (12MO) and month 7 of pregnancy (PREG) for heifers fed a high-protein, high-energy diet (HPHE) during the experimental feeding period. (XLSX)

S5 Table All DEG (FDR<0.05) between experimental feeding period (12MO) and month 7 of pregnancy (PREG) for heifers fed a low-protein, low-energy diet (LPLE) during the experimental feeding period. (XLSX)

S6 Table All DEG (FDR<0.05) between experimental feeding period (12MO) and month 7 of pregnancy (PREG) for heifers fed a high-protein, high-energy diet (HPHE) during the experimental feeding period. (XLSX)

S7 Table Over-represented pathways representing differentially expressed genes between experimental feeding period (12MO) and month 7 of pregnancy (PREG, 20 (HE) and 24 (LE) mo of age) for heifers on a low-protein, high-energy diet (LPHE) during the experimental feeding period. (XLSX)

S8 Table Over-represented pathways affected by transition between experimental feeding period (12MO) and month 7 of pregnancy (PREG, 20 (HE) and 24 (LE) mo of age, low-protein, low-energy diet) for heifers on a high-protein, high-energy diet (HPHE) during the experimental feeding period. (XLSX)

S9 Table Over-represented pathways affected by transition between experimental feeding period (12MO) and month 7 of pregnancy (PREG, 20 (HE) and 24 (LE) mo of age, low-protein, low-energy diet) for heifers on a low-protein, low-energy diet (LPLE) during the experimental feeding period. (XLSX)

S10 Table Over-represented pathways affected by transition between experimental feeding period (12MO) and month 7 of pregnancy (PREG, 20 (HE) and 24 (LE) mo of age, low-protein, low-energy diet) for heifers on a high-protein, low-energy diet (HPLE) during the experimental feeding period. (XLSX)

S11 Table Genes differentially expressed between experimental feeding period (12MO) and month 7 of pregnancy (PREG, 20 (HE) and 24 (LE) mo of age) common to all treatment groups. (XLSX)
